# 

**Published:** 1885-05

**Authors:** 


### CONTENTS:

**Art. I—The Teeth of Children. By Dr. W. George Beers........................1**
**Art. II—The Odontological Society of Great Britain..............................14**
**Art. III—Steam, and its Effects on Rubber and Celluloid when Properly Applied. By Dr. F. W. Seabury............................................25**
**Art. IV—Dentistry in Foreign Countries. By Dr. Charles A. Kingsbury............30**
**Art. V—A Case in Practice. By H. H. Edwards D D. S.,..............................33**
**American Dental Association..................................................38**

### EDITORIAL, ETC.

**California State Dental Law..................................................41**
**A Circular.....................................................................44**

### MONTHLY SUMMARY.

**Regulating Teeth..............................................................45**

**Filling Materials..............................................................47**

